# Multiple Renal Abscesses in a Cow

**Published:** 1880-04

**Authors:** Chas. A. Meyer

**Affiliations:** Consulting Surgeon to Columbia Veterinary College, Hospital Dept.


### III.—MULTIPLE RENAL ABSCESSES IN A COW.

By CHAS. A. MEYER, D. V. S.

Consulting Surgeon to Columbia Veterinary College, Hospital Dept.

On December 12, 1879, I was called to see a blooded cow, *Alderny*, a four-year old, which had given birth to the second calf some weeks previous to my seeing her. The history of the case was as follows :

After she had given birth to her first calf she was taken with what the groom said was paralysis. A " cow doctor " was called in, who treated for such, and who applied turpentine to the loins. She never improved, and through his directions was taken to bull, and after the second parturition the following symptoms were presented to me :

Very much emaciated ; no desire for food; was not able to suckle calf, which was fed by hand; she gave about a quart of milk per diem, which was of a greenish-yellow color, thick, and had a bitter taste ; had a stiff gait in posterior extremities, and no desire to move about ; the tail had lost its power, the urine dribbled away constantly when she was compelled to move, and was thick and ichor-like; there was evidently paralysis of the sphinxter vesicae, due to some organic disease ; the temperature was 102 3-5 ; the respirations were somewhat increased.

My diagnosis was suppurative nephritis, and I recommended the owner to have the animal destroyed. The owner valued the cow highly, and told me she had presented the above symptoms for the past eight months, and wished her to be treated. I had her sent to an infirmary, but gave no medicine; about a week afterwards she suddenly fell dead after having eaten a little green fodder.

The writer, desiring a post-mortem, was immediately informed of her death by the owner, the latter himself being anxious for one. Post-mortem forty-five minutes after death.

On opening the abdomen, no blood seemed to flow ; the lungs were of a healthy appearance, except a small patch of the right lobe, which was emphysematous. The ventricles firmly contracted ; the left auricle and ventricle contained a fibrinous clot, almost white and gelatinous; on opening the aorta, I found the same fibrinous clot, which, on being drawn out, presented a beautiful cast of the aorta and its branches. This clot was proof of prolonged mortal agony. The liver, spleen, and digestive organs presented a healthy appearance ; the kidneys were covered with a dirty, yellowish, infiltrated fat, presenting ecchymoses, near and around the suprarenal capsules. This adipose tissue I removed, and I found a pale appearance of the kidney ; it was almost white, hard and heavy; in cutting through the organ, my knife grated as though cutting through sand, and I was almost compelled to use a saw. The section, when cut, showed an abscess in each lobule of the kidney, whose openings entered the pelvis of the kidney ; each abscess was encased by about an inch of this hard, gritty substance, which tapered gradully towards the pelvis; both kidneys presented the same appearance; the bladder was thickened and contracted, the mucous membrame showed an ichorous, slimy appearance.

The kidneys were unfortunately thrown away through misunderstanding, and therefore I was not able to make a microscopic examination, as I had intended.

